# Experiencing a Severe Weather Event Increases Concern About Climate Change

**DOI:** 10.3389/fpsyg.2019.00220

**Published:** 2019-02-11

**Authors:** Magnus Bergquist, Andreas Nilsson, P. Wesley Schultz

**Affiliations:** ^1^Department of Psychology, University of Gothenburg, Gothenburg, Sweden; ^2^Centre for Collective Action Research, Gothenburg, Sweden; ^3^Department of Psychology, California State University San Marcos, San Marcos, CA, United States

**Keywords:** pro-environmental, environmental concerns, attitudes, extreme weather and climate events, hurrican, repeated-measure

## Abstract

Climate change is primarily driven by human-caused greenhouse gas (GHG) emissions, and may therefore be mitigated by changes to human behavior (Clayton et al., 2015; IPCC, 2018). Despite efforts to raise awareness and concern about climate change, GHG emissions continue to rise (IPCC, 2018). Climate change seems to be at odds with the immediate, present threats to which humans are adapted to cope (Gifford et al., 2009; Schultz, 2014; van Vugt et al., 2014). In contrast to immediate dangers, climate change is typically abstract, large scale, slow and often unrelated to the welfare of our daily lives (e.g., Ornstein and Ehrlich, 1989; Gifford, 2011). But there are moments when the consequences of climate change are readily apparent, such as extreme weather events. In the current paper, we examine the impact of personal experience with an extreme weather event, and the impact of this experience on beliefs about climate change, and intentions to take actions that can help prepare for and mitigate the consequences of climate change.

## Introduction

Experiencing natural disasters can affect people both physically and psychologically. Past research have shown how experiencing natural disasters can affect public health outcomes such as mortality, injuries, infectious diseases, economic impact, and produce a range of psychosocial consequences (Shultz et al., 2005). Natural disasters such as hurricanes, earthquakes, and floods can lead to post-traumatic stress disorder (PTSD), depression, anxiety disorders, and even elevated rates of suicide (Madakasira and O'Brien, 1987; de la Fuente, 1990; Goenjian et al., 1994; David et al., 1996; Krug et al., 1998; Stimpson, 2005).

There is also a line of research focusing on how experiencing extreme weather can affect attitudes and pro-environmental concerns—the “experience-perception link” (Lang and Ryder, 2016). For example, van der Linden (2015) found that experiencing extreme weather events was positively related to environmental risk perception. Similarly, Li et al. (2011) reported that people were more likely to make pro-environmental donations after interpreting local temperature increases as evidence for global warming (see also Joireman et al., 2010).

Some studies do however show that experiencing extreme weather events do not increase environmental concern (e.g., Whitmarsh, 2008). A recent meta-analysis found that self-reported experiences with extreme weather only had a small positive effect on belief in climate change, while experiencing local weather change had a medium sized effect (Hornsey et al., 2016). These findings may be interpreted as suggesting that extreme weather events increase attention to climate change under certain conditions; specifically, when extreme weather events are experienced as abnormal local temperatures (local warming), when extreme weather are temporarily proximal, or when extreme weather events are associated with financial damages (Sicso et al., 2017).

Studies examining the link between personal experience with climate change and subsequent beliefs and actions are just beginning, but there is some circumstantial evidence for an association. For example, Lang and Ryder (2016) used google trends (from 2006 to 2012) and found that search terms related to climate change intensified in the months following tropical cyclones, suggesting that people attributed extreme weather events to global warming. Another study compared student cohorts before vs. after an extreme weather event and found more favorable attitudes toward a climate-protecting politician and higher environmental concerns after the events (Rudman et al., 2013). Similarly, individuals affected by the UK winter flood in 2013/2014 reported stronger negative emotions, greater perceived vulnerability, increased salience of climate change, and higher risk perception compared to a nationally representative sample (Demski et al., 2017). More closely linked to pro-environmental actions, Rochford and Blocker (1991) found that people who perceived the flood in Tulsa, Oklahoma, in 1986 as preventable were more likely to get involved in flood-related activism. Results from a national survey across UK showed that first-hand experience of flooding was positively linked to environmental concern and even greater willingness to save energy to mitigate climate change (Spencer et al., 2011). Importantly, research on the experience-perception link seems to focus on cognitive consequences of experiencing climate change. However, as experiencing natural disasters can cause severe distress (e.g., Goenjian et al., 1994; van Willingen, 2001), we expect that such experiences will also affect emotions. For example, past research has found that U.S. mean temperature anomalies has been positively related to “worry about” climate change (Donner and McDaniels, 2013). Moreover, induced emotions have shown to increase pro-environmental policy acceptance (Lu and Schuldt, 2015), a link mediated by belief in anthropogenic causes natural disasters (Lu and Schuldt, 2016).

Although the experience-perception link of natural disasters has been tested before, past research is limited by measuring (retrospective) self-reported experience, and by using cross sectional designs or cohorts in before vs. after measures (see Reser et al., 2014, for a review). As a result, we know very little about the causal effects in the experience-perception link. For instance, previous beliefs on the causes of climate change may be attributed on the causes of climate-related natural events in order to align with the previous beliefs. In the first of its kind, the present study is a crossover design, recruiting the same participants before and after experiencing a natural disaster. This design enabled us to use repeated-measures in testing if experiencing extreme weather event influences beliefs about climate change, and intentions to take actions. Moreover, as experiencing a natural disaster have shown to result in severe distress (e.g., Goenjian et al., 1994; van Willingen, 2001), we hypothesized that after experiencing a natural disaster people would report stronger negative emotions such as fear when thinking about climate change. Hence, we suggest that when people think about climate change after experiencing extreme weather, climate change will be perceived with stronger negative emotional activation than before. In addition, we wanted to test the experience-perception link, suggesting that experiencing extreme weather positively affects pro-environmental concerns. To test our hypotheses we recruited residents of Florida, USA (without explicitly revealing the aim of the study) before and after hurricane Irma in Eleventh September, 2017.

## Method

### Participants

Using Amazon's Mechanical Turk (MTurk), we exclusively qualified participants that were located in Florida, USA, to take a survey “…*on your beliefs*” in exchange for $0.35. A total of 209 participants answered the first survey (from 8 to 10th of September, 2017) and were invited to take a follow-up survey in exchange for $2 during the 20–28th of September. Although participants were most likely aware of the approaching hurricane when answering the first survey, they had no direct experience of Irma. Therefore, the before- and after-design should validly test the hypothesis of experiencing extreme weather. In line with national and institutional guidelines, approval was not required. All participants gave written informed consent in accordance with the declaration of Helsinki. Because the study involved asking questions concerning a potentially negative experience, we scrutinized the questions making sure they were in accordance with the Swedish Ethical Review Act (2003:460) and that the questions could not, in any way be interpreted as offensive or causing negative affect. Respondents participating in the study were fully informed about the research objectives. Hence, an ethical approval was not required.

### Materials

Both surveys included a core set of measures based on Newman and Fernandes (2016):

- “Willingness to sacrifice” was included to assess participants willingness to reduce own resources as a means to promote pro-environmental outcomes. The scale included 3 items (e.g., *How willing would you be to pay much higher taxes in order to protect the environment? 1: Not at all willing-*−*5: Very willing*), showing acceptable reliability (α_pre_ = 0.92, α_post_ = 0.93).
- “Awareness of consequences” was included to assess participants' perceived danger of anthropogenic climate change. The scale included 6 items (e.g., *In general, do you think that a rise in the world's temperatures caused by climate change is … 1: Not at all dangerous for the environment – 5: Extremely dangerous for the environment*), showing acceptable reliability (α_pre_ = 0.86, α_post_ = 0.88).
- “Personal normative beliefs” was included to assess participants' personal normative aspects about climate change, such as what one “should do” and perceived “responsibility.” The scale included 5 items (e.g., *I worry that the next generation will feel we didn't do enough to prevent climate change. 1: Strongly disagree – 5: Strongly agree*), showing acceptable reliability (α_pre_ = 0.78, α_post_ = 0.83).
- “Progress vs. environment” was included to assess a perceived trade-off between prioritizing human progress or the future environment. The scale included 2 items (e.g., *People worry too much about human progress and not enough about the environment. 1: Strongly disagree – 5: Strongly agree)*, showing low reliability (α_pre_ = 0.59, α_post_ = 0.60).

Participants were also asked how they felt when they “…*think about climate change*,” this was measured with the eight emotions *fear, anger, hope* (R), *sadness, helplessness, guilt, shame, regret* on a scale from “*Very little (1)”* to “*Very much (7)*,” showing acceptable reliability (α_pre_ = 0.90, α_post_ = 0.92). Participants' were also asked about their expectations about the hurricane using two items “*I think that the hurricane Irma will be. 1: Not at all severe*−*5: Extremely severe,” I think that the hurricane Irma will strike. 1: Not at all close to me*−*5: Extremely close to me,”* and one item measured participants' perceived cause of the hurricane “*I think that the hurricane Irma is caused by global warming. 1: Strongly disagree-*−*5: Strongly agree.”* Finally, participants provided demographic measures of age and gender and were given the opportunity to leave a comment.

The second survey included all the items in the first survey. However, when participants were asked about their perceived severity and closeness of Irma in the second survey, we modified the two items as follows: “*I think the hurricane l was…”* (1: *Not at all severe*−5: *Very severe*), and “*I think that Irma stroke…”* (1: *Not at all close to me*−5: *Extremely close to me*). As control- and demographic variables, the second survey also included perceived risks of natural disasters asking participants “*Over the next 20 years in Florida, USA, how likely do you think it is that global warming will cause each of the following? (a) Property damage, (b) Flooded streets, (c) Power outrage, (d) Decreased tourism, (e) Public distress, (f) Deaths, and (g) Public health problems.” Three* items measured if people had taken actions as a consequence of Irma (e.g., *As a consequence of Irma, I have been forced to take actions*). Finally, three single-item questions measured environmental concern (*In general, how concerned are you about the environment?* 1: *Extremely unconcerned*−7: *Extremely concerned)*, political preferences (*I would describe myself as…*1: *Extremely liberal*−7: *Extremely conservative)*, and subjective income *(Please rate your income: Extremely low, Low, Moderate to low, Moderate, Moderate to high, High, Extremely high)*.

## Results

### Sample and Attrition Analysis

One hundred and thirty one participants answered both the first and the second survey. Nine participants were excluded as they were not located in Florida or were outliers in response time when taking the survey. As a result, the final sample consisted of 122 participants (58.2% female, M_age_ = 38, range = 19–73). In political preferences, 34.5% reported being conservative, 41.9% being liberal, and 23.8% in-between conservative and liberal. When reporting subjective income, 39.5% reported extremely low, low, or moderate to low. 49.2% Reported having moderate income, and 10% reported moderate to high, high or extremely high. After experiencing Irma, 82.8% reported that the hurricane stroke very or extremely close to them in space. 87.7% reported taking actions or seeing others take actions as a consequence of Irma. Finally, after Irma, 80.2% reported being concerned about the environment and 82.8% reported that it is somewhat likely or very likely that global warming will cause societal and public health problems in Florida over the next 20 years.

Independent *t*-tests compared participants who answered both surveys to those only answering the first survey on the core set of measures, cause of Irma, emotions, severe, and closeness. No comparison reached significance (all *p*'s > 0.05) indicating that the attrition did not systematically skew the data.

### Pre- and Post-Measures

As our main analysis, we compared answers before vs. after Irma using paired-samples *t*-tests (see Table 1). Results showed that after experiencing Irma, participants reported stronger negative emotions when thinking about climate change compared to before [*t*_(121)_ = 3.00, *p* = 0.003, *d* = 0.30]. After Irma, participants were more willing to sacrifice (pay higher prices, pay higher tax, and accept cuts in standards of living) than before Irma [*t*_(121)_ = 1.99 *p* = 0.049, *d* = 0.17]. However, this effect seems to be driven by the willingness to pay higher taxes to protect the environment after Irma [*t*_(121)_ = 2.45, *p* = 0.016, *d* = 0.24], while the willingness to pay higher prices was not significant and showed a small effect size (*p* = 0.066, *d* = 0.18) and willingness to accept cuts in standards of living was not affected (*p* = 0.909, *d* < 0.01). Participants were more certain that Irma was caused by global warming after experiencing the hurricane compared to before [*t*_(107)_ = 2.4, *p* = 0.018, *d* = 0.23]. There was no evidence for change in personal normative belief before vs. after Irma [*t*_(106)_ = 1.23, *p* = 0.223, *d* = 0.13]. Although a positive tendency, the change for awareness of consequences did not reach statistical significance [*t*_(121)_ = 1.71 *p* = 0.089, *d* = 0.17]. Finally, a marginally significant unexpected negative effect of progress vs. the environment was observed [*t*_(121)_ = −2.0, *p* = 0.050, *d* = −0.21], indicating that participants were less willing to prioritize environmental actions over human progress after Irma (see Table A1, for correlations between all pre-and post-measures). Additional analyses found that neither political preference nor income moderated these repeated-measures effects significantly (all *p*'s > 0.05).

**Table 1:** Effects of experiencing an extreme weather event presented in means and standard deviations for both pre- and post-measures, and *p*-values, effect sizes and confidence intervals for change between pre- and post-measures.

**Measure**	**M**_**T1**_	**SD**_**T1**_	**M**_**T2**_	**SD**_**T1**_	***p*****-value**	**M**_**diff**_	**95% CI M**_**diff**_	***d***_**RM**_
Emotions	4.28	1.26	4.50	1.48	0.003	0.22	0.07, 0.36	0.30
Cause of Irma	3.41	1.25	3.61	1.29	0.018	0.20	0.04, 0.37	0.23
Willingness to sacrifice	3.10	1.13	3.20	1.15	0.049	0.11	0.01, 0.21	0.17
**WILLINGNESS TO…**								
Pay higher prices	3.20	1.15	3.34	1.20	0.066	0.13	−0.01, 0.27	0.18
Pay higher taxes	3.02	1.26	3.21	1.32	0.016	0.18	0.03, 0.33	0.24
Cuts standards of living	3.07	1.24	3.07	1.16	0.909	0.01	−0.13, 0.15	0.00
Awareness of consequences	4.08	0.77	4.17	0.80	0.089	0.09	−0.01, 0.19	0.17
Personal normative belief	3.87	0.82	3.93	0.91	0.223	0.06	−0.04, 0.16	0.13
Progress vs. the environment	3.84	1.00	3.71	1.03	0.050	−0.13	−0.26, 0.01	−0.21

*M_T1_, Mean for time 1; M_T2_, Mean for time 2; SD_T1_, Standard deviation for time 1; SD_T2_, Standard deviation for time 2; M_diff_, Mean difference; 95% CI, 95% Confidence interval; d_RM_, Cohen's d for repeated-measures*.

### Mediation

To explore the mechanisms driving change in willingness to pay higher taxes as a consequence of experiencing Irma, we wanted to test the role of emotions. Change scores (post—pre) were calculated for two variables showing significant change: emotions, and willingness to pay higher taxes. Although not significant, we also included the change scores for personal normative belief, as changes in normative belief may affect the relationship between emotions and willingness to pay higher taxes.

In order to explore possible antecedents of willingness to pay higher taxes, these three variables were correlated. Results showed that change in willingness to pay higher taxes correlated significantly with change in emotions (*r* = 0.20, *p* = 0.03, *n* = 122) and change in personal normative belief (*r* = 0.19, *p* = 0.045, *n* = 107).

To further explore our main proposition, that perceiving Irma would increase willingness to pay higher taxes, we tested if the relationship between increased negative emotions and increased willingness to pay higher taxes was mediated by change in personal normative belief. Using the software *Process* 2018 in *SPSS* we ran a mediator analysis (Model 1). We entered change in personal normative belief as a mediator in the path between change in negative emotions and change in willingness to pay higher taxes. Results showed a significant model [*F*_(3,103)_ = 3.06, *p* = 0.03, *R*^2^ = 0.08]. The model revealed a significant direct effect of emotions on willingness to pay higher taxes (β = 0.21, *t* = 2.10, *p* = 0.04, 95% CI [0.01,0.40]). However, neither the effect of personal normative belief (β = 0.19, *t* = 1.24, *p* = 0.22, 95% CI [−0.12,0.50]), nor the interaction term (β = 0.06, *t* = 0.35, *p* = 0.72, 95% CI [−0.26,0.37]) were significant.

**Figure 1:**
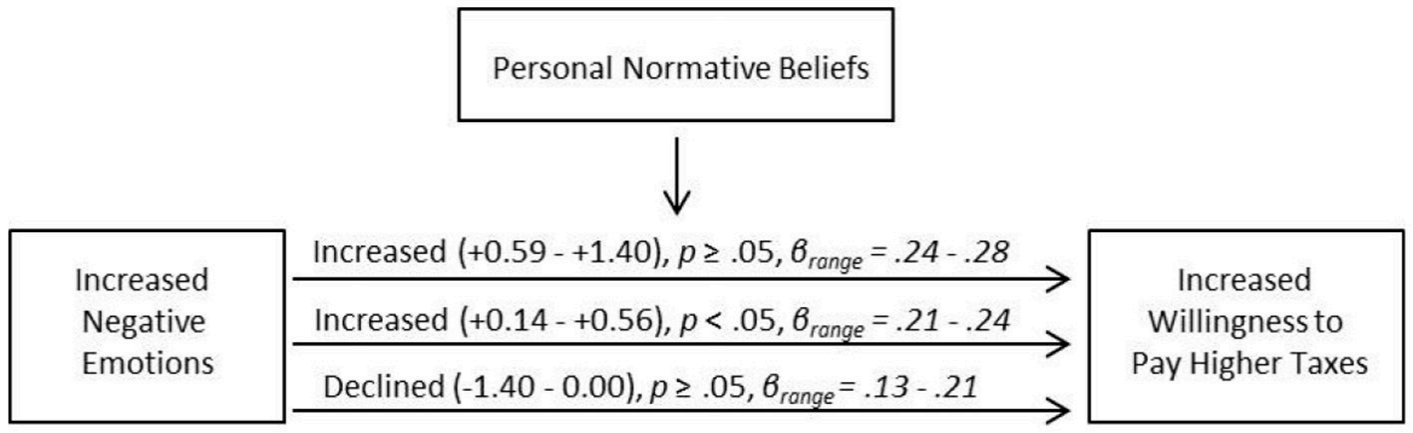
Mediational model for positive relationships between increased negative emotions and increased willingness to pay higher taxes at given levels of change in personal normative beliefs.

In further exploring these data, the Johnson-Newman technique revealed that the effect of emotions on willingness to pay higher taxes was significantly mediated by change in personal normative belief within the regions of 0–0.56 (see Table A2). This suggests that for participants who expressed lower personal normative beliefs after Irma (−1.4–0), the relationship between negative emotions and willingness to pay higher taxes was non-significant. Only for participants showing an increase of personal normative belief (+0.14–+0.56) was strengthened negative emotions positively related to increased willingness to pay higher taxes. It should however be noted that for participants with stronger increase in personal normative belief (+0.59–1.40), the relationship between negative emotions and willingness to pay higher taxes was not significant. However, the descriptive tendency was that stronger increase in personal normative beliefs related to higher beta-values between negative emotions and willingness to pay higher taxes, (see Figure 1). This implies that when experiencing Irma induced heightened negative emotions while at the same time not decreasing personal normative beliefs (for example, worrying that we did not do enough to prevent climate change for the next generation) respondents also show stronger willingness to pay higher taxes for the sake of the environment.

Taken together, this suggests that in order for extreme weather experiences to result in pro-environmental actions, people need to feel that this experience was negative and think that they ought to do something about it.

## Discussion

Climate change is a difficult threat for humans to cope with in a constructive manner. The problems associated with climate change are abstract and large scale, and they differ from the more immediate threats that humans are adapted to respond to. The abstract and long-term characteristic of climate change can also promote psychological rationalizations for not taking action to mitigate and prepare (Gifford, 2011). But there are moments when the consequences of climate change are readily apparent. In the present research, we examine the impact of personal experience with an extreme weather event, and the impact of this experience on beliefs about climate change, and intentions to take actions that can help prepare for and mitigate the consequences.

In a unique before and after study, we examined changes in beliefs about climate change and intentions to support or take actions before and after hurricane Irma. We found that respondents expressed stronger negative emotions toward climate change, were more certain that the hurricane was caused by global warming, and were more willing to pay higher taxes after experiencing Irma. These results support previous research on self-reported experiences (Reser et al., 2014) suggesting that experience of extreme weather events influences beliefs about climate change and intentions to mitigate its effect.

Among the variables measuring willingness to sacrifice, respondents were more willing to pay higher taxes after Irma, while there was no change in willingness to accept cuts in standards of living. One explanation for this could be that while it may seem reasonable to pay higher taxes to support mitigation and adaptation, accepting cuts in standard of living while coping with restoring the effects of the hurricane is less reasonable. A similar explanation can be given for the results on the “progress vs. environment,” which revealed a negative effect, showing that participants were more likely to prioritize human progress over the environment after Irma. Human progress may in this case be interpreted as restoring societal functions after Irma. That is, for people living in areas damaged by natural disasters, restoration may be perceived as more important than prioritizing pro-environmental actions. As some factors are more strongly related to climate change adaptation behaviors than others (van Valengoed and Steg, 2019), we would like to encourage future research to explore under which conditions extreme weather experience cause people to prioritize climate change adaptation behaviors over human progress and vice versa.

In an explorative mediation analysis, we found that heightened negative emotions as a consequence of experience extreme weather events have a direct effect of peoples' willingness to pay higher taxes. Importantly, we also found a mediating effect showing that this relationship was affected by if people though they ought to do something about the climate. More specifically, strengthened negative emotions were related to increased willingness to pay higher taxes only for people who showed stronger or unchanged personal normative beliefs. This finding has practical implications as it suggests that policy supports (i.e., pay higher taxes) as a result of experiencing extreme weather events, depends both on peoples emotional response and normative considerations (for example, thinking the next generation feels that “we did not do enough to prevent climate change”).

One possible limitation when drawing conclusions of the results is that observed changes may partially be attributed to media coverage about the hurricane. Since no control group in areas not affected by the hurricane was used, the influence of such media exposure cannot be ruled out. We encourage future research, examining the experience-perception link, to add a control group which may be exposed to media reports, but not having first-hand experience of the extreme weather.

Future research should investigate other types of extreme weather events in order to corroborate the results in this study. Extreme weather events come in many shapes; flooding, hurricanes and drafts can all be observable potential climate impacts that may influence beliefs and intentions. The relationships between different types of weather events and peoples' reaction to these in terms of connection to climate change may differ however, and should be studied using before and after designs.

## Conclusion

In a unique repeated-measures design, we examined the experience-perception link of climate disasters, and conclude that experience matters. Experiencing the hurricane Irma intensified Floridians negative emotions toward climate change, strengthened their beliefs in that Irma was actually caused by global warming, and fostered a willingness to sacrifice to reach environmental solutions.

## Author Contributions

MB developed the surveys in discussion with AN. MB collected and analyzed the data with input from AN and PS. All authors contributed in writing the manuscript and all authors approved the final version of the manuscript for submission.

### Conflict of Interest Statement

The authors declare that the research was conducted in the absence of any commercial or financial relationships that could be construed as a potential conflict of interest.
