# Supplementary material for: Experiencing a Severe Weather Event Increases Concern About Climate Change
Source: Front Psychol. 2019 Feb 11;10:220. doi: 10.3389/fpsyg.2019.00220 (PMC6378300; doi:10.3389/fpsyg.2019.00220)
Supplement: Supplementary file 1 [file Data_Sheet_1.docx]

**Appendix A**

Table 1. *Correlational matrix including pre- and post-measures of “Willingness to sacrifice, “Awareness of consequences”, “Personal normative beliefs”, “Progress versus environment”, Emotions, and ratings of how severe and close whey were to the hurricane Irma, and the attributional cause of the hurricane Irma.*

|  | 1 | 2 | 3 | 4 | 5 | 6 | 7 | 8 | 9 | 10 | 11 | 12 | 13 | 14 | 15 |
| --- | --- | --- | --- | --- | --- | --- | --- | --- | --- | --- | --- | --- | --- | --- | --- |
| 1.Willingness to pay higher taxes:  pre-measure |  |  |  |  |  |  |  |  |  |  |  |  |  |  |  |
| 2.Willingness to pay higher taxes:  post-measure | .80** |  |  |  |  |  |  |  |  |  |  |  |  |  |  |
| 3. Awareness of consequences:  pre-measure | .53** | .48** |  |  |  |  |  |  |  |  |  |  |  |  |  |
| 4. Awareness of consequences:  post-measure | .57** | .58** | .75** |  |  |  |  |  |  |  |  |  |  |  |  |
| 5. Personal normative belief:  pre-measure | .64** | .58** | .72** | .66** |  |  |  |  |  |  |  |  |  |  |  |
| 6. Personal normative belief:  post-measure | .62** | .63** | .56** | .72** | .84** |  |  |  |  |  |  |  |  |  |  |
| 7. Progress vs. the environment:  pre-measure | .59** | .50** | .52** | .55** | .65** | .71** |  |  |  |  |  |  |  |  |  |
| 8. Progress vs. the environment:  post-measure | .51** | .47** | .50** | .60** | .68** | .71** | .72** |  |  |  |  |  |  |  |  |
| 9. Emotions:  pre-measure | .63** | .63** | .59** | .60** | .65** | .62** | .56** | .57** |  |  |  |  |  |  |  |
| 10. Emotions:  post-measure | .62** | .69** | .54** | .70** | .66** | .73** | .56** | .61** | .84** |  |  |  |  |  |  |
| 11. Cause of Irma:  pre-measure | .57** | .49** | .59** | .57** | .56** | .55** | .49** | .51** | .60** | .60** |  |  |  |  |  |
| 12. Cause of Irma:  post-measure | .52** | .52** | .53** | .67** | .56** | .62** | .45** | .53** | .59** | .71** | .76** |  |  |  |  |
| 13. Severity of Irma:  pre-measure | .08 | .06 | .31** | .33** | .19* | .13 | .17 | .15 | .17 | .08 | .16 | .13 |  |  |  |
| 14. Severity of Irma:  post-measure | .12 | .18 | .19* | .22* | .24** | .20* | .09 | .12 | .15 | .26** | .24** | .34** | .32** |  |  |
| 15. Closeness of Irma:  pre-measure | .17 | .09 | .24** | .24** | .15 | .10 | .20* | .11 | .13 | .09 | -.03 | .10 | .33** | .05 |  |
| 16. Closeness of Irma:  post-measure | .24** | .16 | .11 | .23* | .13 | .08 | .13 | .00 | .09 | .11 | -.02 | .09 | .14 | .00 | .39** |

*Note.*  * = *p* < .05, **** = *p* > .01

Table 2. *Full output for the relationship between change in*

*negative emotions and change in willingness to pay higher*

*taxes at given levels of change in personal normative beliefs.*

| Change in personal normative belief | *Β* for negative emotions and  willingness to pay higher taxes | *p* for negative emotions and  willingness to pay higher taxes |
| --- | --- | --- |
| -1.40 | .13 | .63 |
| -1.26 | .14 | .58 |
| -1.12 | .14 | .52 |
| -.98 | .15 | .46 |
| -.84 | .16 | .39 |
| -.70 | .17 | .31 |
| -.56 | .17 | .24 |
| -.42 | .18 | .17 |
| -.28 | .19 | .11 |
| -.14 | .20 | .07 |
| -.06 | .20 | .05 |
| 0 | .21* | .04 |
| .14 | .21* | .03 |
| .28 | .22* | .03 |
| .42 | .23* | .03 |
| .56 | .24* | .04 |
| .59 | .24 | .05 |
| .70 | .25 | .07 |
| .84 | .25 | .09 |
| .98 | .26 | .12 |
| 1.12 | .27 | .15 |
| 1.26 | .28 | .18 |
| 1.40 | .28 | .20 |

*Note. ** significant region.
